# Global Perspective on the Management of Peyronie's Disease

**DOI:** 10.3389/frph.2022.863844

**Published:** 2022-06-09

**Authors:** Gabriel Veber Moisés da Silva, Francisco Javier Dávila, Tiago Elias Rosito, Francisco E. Martins

**Affiliations:** ^1^Department of Urology, Hospital de Clínicas de Porto Alegre, Universidade Federal do Rio Grande do Sul (UFRGS), Porto Alegre, Brazil; ^2^Department of Urology, University of Lisbon, School of Medicine, Centro Hospitalar Universitario Lisboa Norte (CHULN), Lisbon, Portugal

**Keywords:** grafting, penile, curvature, deformity, plaque, prosthesis, Peyronie's disease, penile reconstruction

## Abstract

**Introduction:**

Peyronie's disease is the disease that results in an alteration in the curvature of the penis, which can lead to a shortening of length, pain in erection, or difficulties in penetration, thus leading the patient to psychological alterations due to loss of functionality such as aesthetic alteration. That is why there are several studies to define the best form of treatment, which currently continues to be the first choice surgical treatment.

**Objective:**

We present the most recommended therapies for Peyronie's disease and suggest an algorithm as a guide to direct therapy.

**Methods:**

We used the PubMed platform to review the literature related to Peyronie's disease. Various editorials were reviewed as well as original articles and reviews focusing on the various treatments as well as their indications and results.

**Results:**

Peyronie's disease in which conservative or drug treatment does not have a response, surgical treatment with corporoplasty, penile prosthesis implantation or both may be indicated. Corporoplasty refers to both the plication of the tunica albuginea as well as the incision of the tunica with the placement of a graft. An accurate history should always be carried out to identify erectile dysfunction as well as to be able to guide you on the repercussions of the treatment. If refractory erectile dysfunction is present, placement of a penile prosthesis with or without further adjunctive straightening maneuvers is recommended. We reviewed the indications, advantages, disadvantages, and results of the available techniques, and proposed a surgical treatment algorithm.

**Conclusion:**

Penile shortening procedures are usually indicated in curvatures <60°, in penises with adequate length. Partial excision/incision and grafting are indicated for curvatures >60°, hourglass or hinge deformities, and short penises, if the patient's erectile function is adequate. The presence of “borderline” erectile function and/or ventral curvature tilts the choice toward shortening procedures, and refractory erectile dysfunction is an indication for penile prosthesis placement. An accurate risk/benefit assessment of the individual patient as well as meticulous patient counseling are critically important.

## Introduction

In 1,743 Francois Gigot de la Peyronie, was the first person to describe the Peyronie's disease (PD) characterized by a wound healing disorder that leads to an alteration in the tunica albuginea of the penis, resulting in a plaque formation due to the deposition of collagen and fibrin occurring after an injury to the penis (1–3).

The underlying pathophysiology remains not fully understood and this lack of knowledge leads to an inability to prevent the starting and the progression of the disease, making PD a challenging disorder to treat. It is a consensus among the majority that the initial injury is due to some form of penile trauma, which, in susceptible patients, triggers a cascade of events leading to the development of Peyronie's disease. Normal penile architecture is essentially lost consequently. This leads to the formation of Peyronie's plaque, which is characterized by disorganization of collagen and elastin fibers, producing an asymmetric expansion of the corpora cavernosa with the characteristic deformity of the erect penis. This cascade of events appears to involve free oxygen radicals, oxidative stress, nitric oxide, myofibroblasts, TGF-β1, and fibrotic gene expression. Regarding the impact of genetic factors predisposing to PD and other fibrotic diseases, this remains unclear. The first evidence that a genetic factor could influence PD was in 1982, correlating PD and Dupuytren's disease (4). Since then, much progress has been made to establish the links between fibrotic disorders, however, the specific genes are not currently known. Patterns of gene expression in families with PD and Dupuytren's disease are similar, particularly regarding collagen degradation, ossification, and myofibroblast differentiation. A study comparing gene-expression profiles of PD patients with those of DD patients found a series of 15 genes that were upregulated in the PD plaque vs. the normal tunical albuginea, the ones most prominently increased were matrix metalloproteinases (MMPs) involved in collagen breakdown, specifically MMP-2 or MMP-9 in one-half of the PD plaques (4). Future studies on these mechanisms are likely to be the keyway to further elucidate the exact mechanism behind the disease development (2–7).

The prevalence of PD is estimated from 3 to 9% and varies widely depending on the population being studied (1). These data remain limited and variable due to the lack of standardized studies. Most of these studies estimate the prevalence of PD by studying different age groups and specific subgroups of individuals, such as those seeking care for screening for prostate cancer and are not based on population studies. Brazilian study evidence at a total of 954 men undergoing a routine screening for prostate cancer and demonstrated the prevalence of PD of 3.67% (8). A German study including 8,000 men aged 30–80 years who were sent a questionnaire was able to demonstrate a prevalence of 3.2% for the presence of palpable penile plaque (9). Another example is a US web-based survey conducted among the general population of men aged 18 years or older demonstrated that 0.7% (57/7711) had definitive PD and 11.0% (850/7711) had probable PD, all based on self-reported symptoms without specialized evaluation (10).

Most diagnoses are made in the fifth decade of life, with an increasing incidence after the age of 50 years (9). Another associated condition that has been studied is diabetes mellitus (11). Some studies show a prevalence of up to 33.2% of DM in patients with PD (12). The mechanism by which diabetes increases the incidence of Peyronie's appears to be the induction of tissue alteration leading to tissue remodeling after microtraumas during sexual intercourse. Added to this is the increased risk of erectile dysfunction, which makes the penis less rigid and more susceptible to trauma during sexual intercourse. In general, men with Peyronie's disease have more erectile dysfunction, as shown in several studies. Other associations are being studied, namely, testosterone deficiency, prostate cancer, and radical prostatectomy (13). Peyronie's disease could also be related to other concurrent systemic collagen disorders such as Dupuytren's disease, a condition that affects the palmar fascia of the hand and could be present in 10–20% of men with PD, and Ledderhose disease, which affects the plantar aponeurosis of the foot (2, 13).

Symptoms most frequently reported by patients with Peyronie's disease include pain, deformity, palpable plaque, erectile dysfunction, and/or inability to maintain penetration for intercourse. Patients may present pain at the time of palpation of the plaque, onset of erection, even with a flaccid penis; however, pain predominates in the acute phase and during sexual intercourse. Usually, the pain resolves during the stable phase of the disease, but it may remain in some men, especially during erection. The perception of penile curvature is the most distressing symptom associated with the disease, as well in case the curve is in the dorsal region, with prominence along the axis and less than 60°, the ability to maintain sexual activity could still be possible.

## Patient Evaluation and Diagnosis

Currently, a standardized approach to assess the Peyronie disease does not exist, and diagnostic tools for measurement or assessment are limited (14, 15).

The initial evaluation includes a detailed medical and sexual history, onset and stage of the disease (acute/active vs. chronic/stable), precipitating event (if known), penile pain, number, location and consistency of plaques, nature and extent of curvature, and overall subjective level of sexual ability (14). Active disease is defined as the recent onset of symptoms, painful erection, and progressive worsening of the penile curvature. Resolution of pain and stability of the curvature normally occurs within 12–24 months after the onset of symptoms. The well-accepted criterion of stable disease is having no progressive symptoms for at least 3 months.

A more comprehensive physical examination should be done to identify other sites of potential fibrotic changes, like hands and feet. The patient should be asked whether there is any personal or family history of other fibrotic disorders including Dupuytren's and Ledderhose disease and tympanosclerosis.

The sexual function and penile rigidity may be further assessed using the International Index of Erectile Function (IIEF) and an Erection Hardness Score numerical scale, respectively. An important question should be made: “Would you be able to perform a complete sexual intercourse if you did not have any penile deformity?”.

It is important to evaluate thoroughly four important characteristics of the disease: (1) penile curvature, (2) indentation/hourglass deformity, (3) penile plaque, and (4) penile shortening. These 4 aspects of the disease will guide the final treatment plan. Penile Doppler ultrasound is indicated for any PD patient who desires “invasive” treatment. It is critical to obtain low-threshold information preoperatively on those who report significant curvature, palpable penile plaques, and no recent erections.

Additional questions should investigate the impact on quality of life and distress caused by Peyronie's disease. Hellstrom et al. validated a specific questionnaire, the Peyronie's Disease Questionnaire (PQD), which is a 15-question self-report instrument composed of 3 essential domains, (1) psychological and physical symptoms, (2) penile pain, and (3) symptom annoyance, that can enable clinicians to assess and monitor symptoms and to quantify the psychosexual impact of PD (14, 15).

The standard examination is palpation of the flaccid penis on stretching for identification of the number, location, and extension of the plaques. This maneuver is performed by grasping the glans and pulling it at a 90-° angle away from the pubis. The stretched penile length (SPL) is measured from the pubis to the corona dorsally.

Assessment of penile deformity in the erect state is an important step toward the therapeutic decision. Photographs taken at home, as described by Kelami, should include self-photography of the erect penis from above, sides, and end face. This can be a useful tool to get a general impression of the PD. However, because it cannot precisely represent and measure a three-dimensional deformity, its value has been controversial (15). The most accurate method is an induced erection after an office vasoactive injection. This allows the objective measure of the penile length, the grade of the curvature, as well as identification of additional penile deformities, like hourglass or hinge defects. The use of a routine penile duplex ultrasound with pharmacological stimulation can be useful. The goal is to obtain penile vascular flow parameters and assess plaque characteristics (16, 17). The penile Doppler US scan is critical in the evaluation of “high-risk” Peyronie's disease patients as 23–60% of these patients have veno-occlusive dysfunction, 19–25% will show curvatures > 60° and all candidates for plaque incision and grafting procedures because these may place them at risk for erectile dysfunction after surgery (15–17). These criteria are critical to the decision process for the potentially surgical patient.

## Disease Management

Conservative medical treatment is indicated in the active stage of the disease. Current therapies include oral substances, topical treatments, injection of intralesional active agents, and local physical devices, like extracorporeal shock wave therapy [ESWT], traction, and vacuum devices (18, 19).

### Conservative Treatment

#### Oral Medications

Non-steroidal anti-inflammatory drugs (NSAIDs) may be offered to patients in active-phase PD for pain management, which is usually present in this phase.

Pentoxifylline decreases transforming growth factor (TGF) beta-1-mediated fibrosis, prevents the deposition of collagen type I and reduces calcification in experimental animals. Some studies showed improvement in penile curvature and plaque volume with the use of pentoxifylline 400 mg two or three times a day associated with antioxidants and/or intralesional therapy (19, 20).

Potassium para-aminobenzoate (POTABA) is an antifibrotic agent of the vitamin B complex and is thought to reduce the formation of collagen. One trial showed a benefit compared with placebo in reduction of plaque size and/or penile curvature (21). However, because it carries a significant cost, is difficult to consume (24 tablets daily), being known for its gastrointestinal side effects and there is little evidence of benefit in placebo-controlled trials, its use is not recommended.

Vitamin E is one of the oldest oral treatments for PD. It is a potent antioxidant that is thought to limit oxidative stress of reactive oxygen specimens. Despite being the most prescribed oral treatment, several well-designed placebo-controlled studies show no benefits, (22) and its use is not recommended.

Colchicine has multiple mechanisms of action, such as inhibiting cell mitosis, mobility and adhesion of leukocytes, and stimulates the production of collagenase. Because of its relatively common gastrointestinal side effects and potential for bone marrow suppression, its use is not recommended to treat PD.

Tamoxifen, a selective estrogen receptor modulator, can induce the production of TGF- β, inhibiting the inflammatory response and preventing further fibrogenesis. In a randomized, double-blind trial of tamoxifen versus placebo (*n* = 25), the proportion of patients who reported a reduction in curvature was similar in both groups (46 vs. 42%) (23). Its use is not recommended to treat PD.

Phosphodiesterase type 5 inhibitors, a well-recognized treatment for erectile dysfunction, have also been suggested as a treatment for PD, by increasing the levels of cGMP, PDE5 can inhibit the synthesis of collagen, acting as an antifibrotic agent. A study by Chung et al. demonstrates that a low-dose regimen of tadalafil (2.5 mg daily over a 6-month period) is a safe and effective treatment option in septal scar (24). It can also be used in conjunction with penile extracorporeal shockwave therapy (ESWT), improving IIEF, and quality-of-life (25). Although evidence of possible benefits, PDE5 is still not recommended for the treatment of Peyronie's disease.

Currently, no oral agent has been shown in placebo-controlled trials to result in clinically meaningful improvement in curvature. As a result, the only oral medication recommended by the AUA guideline on PD is NSAIDs to reduce pain.

#### Intralesional Injection

Intralesional verapamil is thought to influence fibroblast metabolism by increasing collagenase activity and concurrently decreasing collagen production (20). In a systematic review including four prospective studies of patients with PD, verapamil improved penile curvature, plaque size, and penile pain (26, 27). Due to the use of different treatment protocols and conflicting results, its use as a routine treatment option is still controversial (20, 26, 27).

Collagenase *Clostridium histolyticum* (CCH), the first US Food and Drug Administration (FDA)—approved drug for the treatment of PD, is produced by the bacterium *C. histolyticum* and selectively degrades collagen types I and III in connective tissues. The original treatment protocol consists of two injections of 0.58 mg of CCH 24–72 h apart every 6 weeks for up to four cycles. Modified protocols are being studied but there is still no consensus about the optimized dosage (28).

Gelbard et al., in the IMPRESS trial, examined CCH treatment through a maximum of 8 injections of 0.58 mg in subjects with PD. Following 52 weeks of treatment, men treated with CCH had a 34 percent improvement in curvature compared with 18% in the placebo group. Three men experienced corporeal rupture, that were surgically repaired successfully (28).

Usually indicated in patients in the stable phase of the disease, 3 studies analyzed CCH use in patients in the acute phase. All of them demonstrated that toxin treatment was also useful in reducing the curvature in this phase. Only one study was prospective, with a small number of patients (18, 29, 30).

Abdel Raheem et al. and Capece et al., in 2 prospective non-randomized studies with 53 and 135 patients, respectively, studied CCH treatment in a modified short protocol application (3 cycles of 0.9 mg each at 4-week intervals). They demonstrated a significant reduction in penile curvature, with lower costs and a shorter duration of treatment (31, 32).

In conclusion, CCH is a safe and established treatment. The ideal candidate for therapy remains elusive. It seems that the man with the greatest chance of curvature improvement has curvature between 30° and 60°, duration of disease >12 months, good baseline sexual function (IIEF>17), and no calcification. For a better understanding, more prospective, controlled studies with a greater number of patients are needed (33).

#### Topical Therapy

Several studies have evaluated the effectiveness of topically applied agents for the treatment of PD. The rationality is that topical application avoids the pain and trauma of intralesional injection therapy. Topical verapamil gel was studied in a small, randomized placebo-controlled trial and it was better than the placebo in eliminating pain on erection, decreasing plaque size (84.7 vs. 55%), decreasing curvature (61.1 vs. 43.6%), and improving erection quality in patients with PD (34). However, it is uncertain whether topical therapy influences penile plaques, as topically (transdermal) administered verapamil gel has been shown to be ineffective in achieving therapeutic levels into the tunica albuginea (25). Another topically applied medication studied was liposomal recombinant human superoxide dismutase which did not demonstrate significant effects upon plaque size or penile curvature (35).

Although possibly promising, at this time no topically applied agent has been established as an effective PD treatment.

#### Extracorporeal Shock Wave Therapy

The mechanism of action involved in ESWT for PD is unclear. There are two purported hypotheses: shock waves cause direct damage to the penile plaque, and ESWT increases the vascularity of the targeted area by generating heat, which leads to the induction of an inflammatory reaction, resulting in lysis of the plaque and removal by macrophages (36).

In the first prospective randomized double-blind placebo-controlled clinical trial evaluating ESWT for the treatment of PD, 50 patients randomized to ESWT demonstrated improvements in pain, IIEF-5 score, and mean QoL score. Plaque size and penile curvature were not significantly different between groups (37). Another study conducted by Hatzichristodoulou et al. showed the same results: reduction in pain in the ESWT group (85 vs. 48%, *p* = 0.013) and no changes in plaque size or penile deviation (26).

In conclusion, randomized controlled trials examining the efficacy of ESWT do not show a benefit in curvature reduction, plaque size, or erectile function, although a statistically significant reduction in penile pain has been observed. These data remain limited and variable due to the lack of standardized studies, lack of strict inclusion criteria regarding disease duration, and not standardized method of EWST protocol application. At this time, ESWT should be offered to improve penile pain.

#### Penile Traction

Controlled stretching of the penis by a device is a non-invasive, non-surgical treatment modality that induces cellular proliferation, triggers scar modeling, and reorients the collagen fibrils, in a process known as mechanotransduction.

The first study of penile traction conducted by Levine et al. demonstrated in 10 men efficacy in reducing curvature up to 45° and in increasing penile length 0.5–2 cm (38). In this study, traction was applied 2–8 h per day for 6 months. There were no serious adverse events.

Martínez-Salamanca et al. conducted a study on 55 patients with Peyronie's disease in the acute phase who underwent penile traction compared with 41 patients also in the acute phase who did not. Traction was applied 6–9 h per day (mean duration use was 4.6 h per day), preventing its use during sleep, for 6 months. The mean curvature decreased from 33° at baseline to 15° at 6 months and 13° at 9 months with a mean decrease of 20°in the traction group. The need for surgery was reduced in 40% of patients who would otherwise have been candidates for surgery and simplified the complexity of the surgical procedure (from grafting to plication) in one of three patients. The overall satisfaction rate was 85% (39).

The most recent developed device was presented in a randomized, single-blind, controlled trial conducted by Ziegelman et al. The main advantage was a duration of applied traction: 30–90 min per day for 3 months. There was an improvement of 11.7° in the curvature in 3 months in the device group compared with a worsening of 1.3° in the control group (no traction) (*p* < 0.001). The therapy group also showed significant improvement over controls in penile length (1.5 vs. 0 cm, *p* < 0.001). There were no significant adverse events (40).

The main concerns related to the use of penile traction are its long application time, difficulty in its use (fixation on the glans), local discomfort, and conflicting data in the studies. Traction therapy has the potential to be an effective non-surgical treatment to recover lost length, reduce curvature, and enhance girth.

#### Vacuum Therapy

Although the application of vacuum erection devices is considered safe, it would not induce the desired physical changes induced by mechanotransduction. There is no recommendation regarding vacuum therapy as a treatment for PD.

### Surgical Treatment

Surgical management is indicated for patients who have remained with PD for more than 12 months and present a significant penile deformity with impaired sexual function. The aim of the surgery is to correct the curvature and allow penetrative intercourse. Other precipitants that indicate surgery are extensive penile calcification and/or plaques, failed conservative medical treatment, and patient preference.

As mentioned before, it is essential to document the disease before performing the surgery (penile anatomy, location and size of the plaques, degree of penile deformity, length of the penis, and presence of erectile dysfunction). Before surgery, the objectives and risks of surgery should be discussed, such as persistent or recurrent curvature, reduction in the length of the erect penis, decrease in rigidity, and decrease in sexual sensation. The goal is to make the penis “functionally straight.”

Each surgical approach must be chosen depending on the complexity of the PD as well as the different degrees of involvement, so the selection of the appropriate surgical procedure is the most important factor that determines the success of the treatment (41).

Surgical options include tunica albuginea treatment such as tunica shortening (eg, plication), tunica lengthening (eg, grafting), or penile prosthesis implantation in patients with associated severe erectile dysfunction (combined or not with adjuvant procedures to allow for resolution of deformity) (Table 1).

**Table 1:** Options of surgical treatment.

Tunical shortening procedures	Nesbit plaque excisional procedure Yachia plaque incisional procedure Essed-Schroder plaque plication procedure Lue 16-dot plication procedure
Tunical lengthening/grafting procedures	Partial incision and grafting (PIG) Partial excision and grafting (PEG)
Penile prosthesis implantation (associated procedures if necessary)	Plication sutures Tunical incision with/without grafting (PIG) Penile length restoration Manual modeling

#### Tunical Shortening Procedures

Shortening the longest and convex side of the penis should be considered in patients with adequate penile length, no complex deformities, and no severe curvature with good erectile function. It also could be offered as a treatment option for patients with severe curvatures (>60°) and adequate erectile function and penile length who do not desire more complex procedures.

The advantages of these surgical approaches in addition to a shorter surgical time with minimal effect on the erection include good cosmetic results and effective straightening. Disadvantages include shortening and lack of correction with altered aesthetics of an hourglass or hinge. Nesbit et al. in 1965 were the first to describe the procedure (42). He performed an elliptical excision removal of a portion of the tunica albuginea on the contralateral side of the defect with permanent suture primary closure (43). Yachia modified the technique using a Heineke-Mikulicz principle (a full-thickness vertical incision in the tunica albuginea contralateral to the area of greatest curvature and transversely closed without removal of tunica albuginea) (44).

In 1985, Essed and Schroeder described the plication technique in the form of eight with non-absorbable sutures, thus initiating imbrication techniques in which they did not need to incise the tunica albuginea to correct PD (45). There is another variation of the technique without incision, which is the 16-point technique. The tunica albuginea is plicated with permanent suture using an extended Lembert-type suture placement following four dots per plication (46).

Results and satisfaction rates are both similar to the incision/excision procedures.

#### Tunical Lengthening Procedures

**Figure 1:**
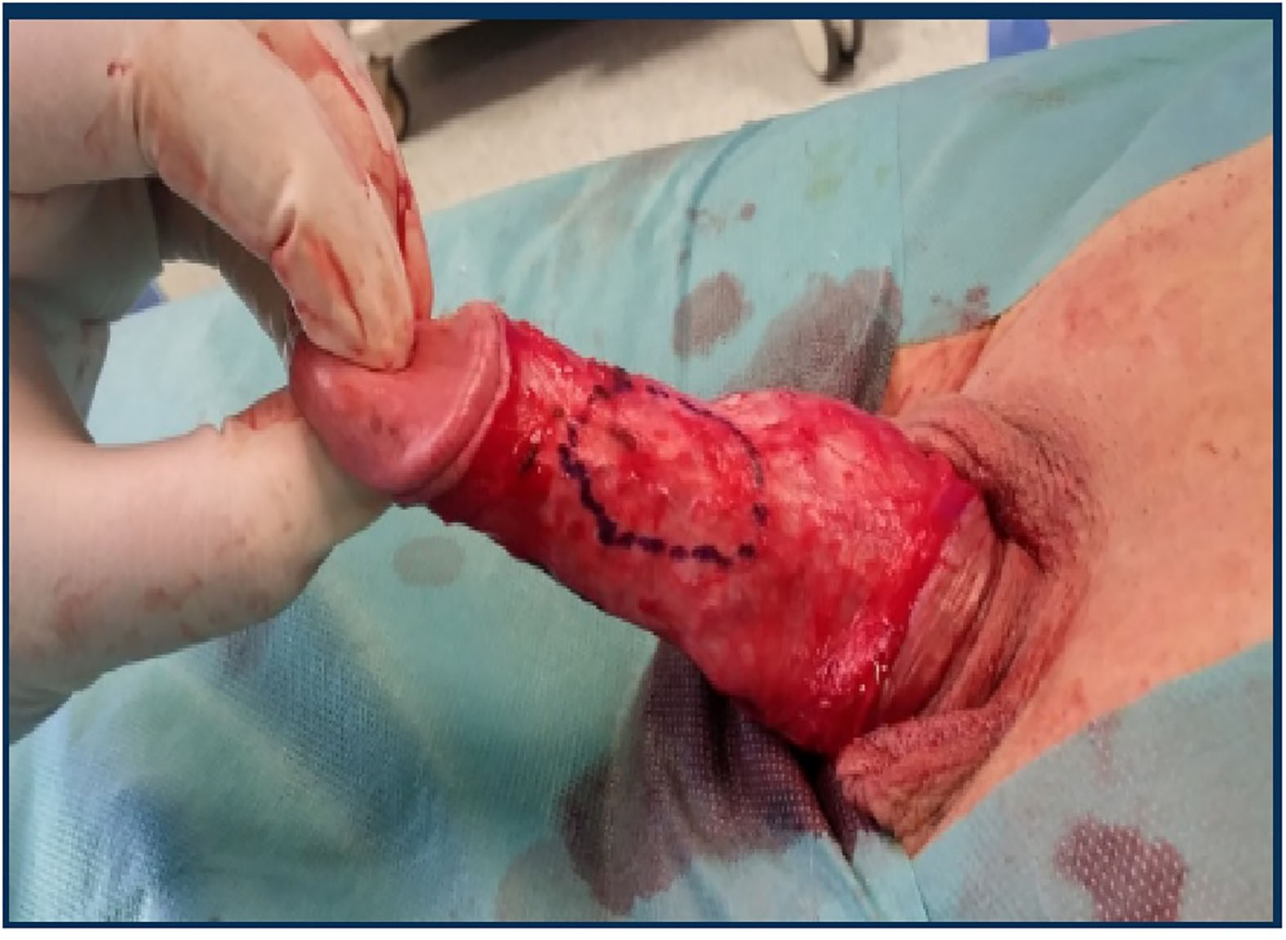
Plate delimitation.

**Figure 2:**
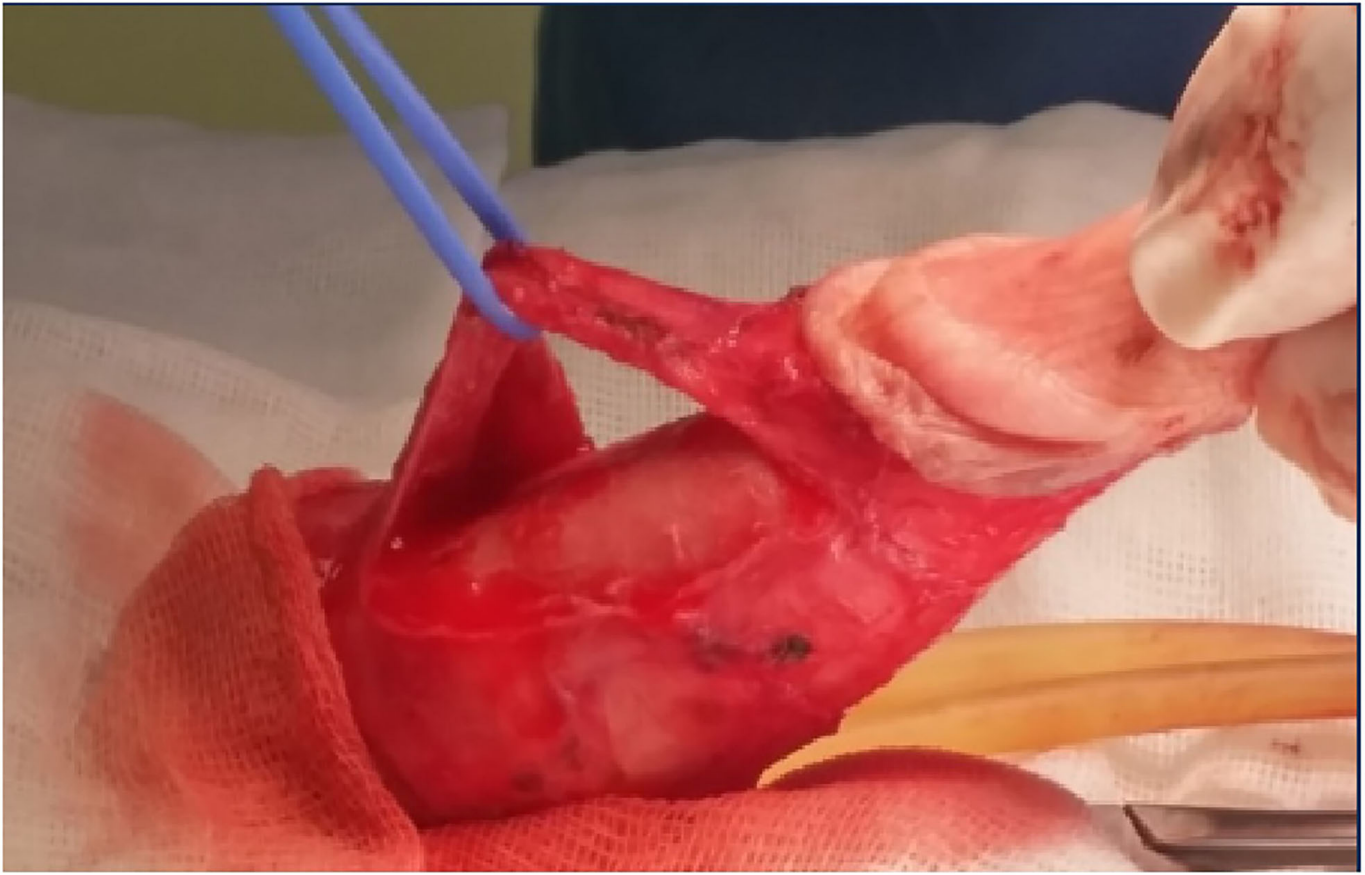
Isolation of neurovascular bundle.

**Figure 3:**
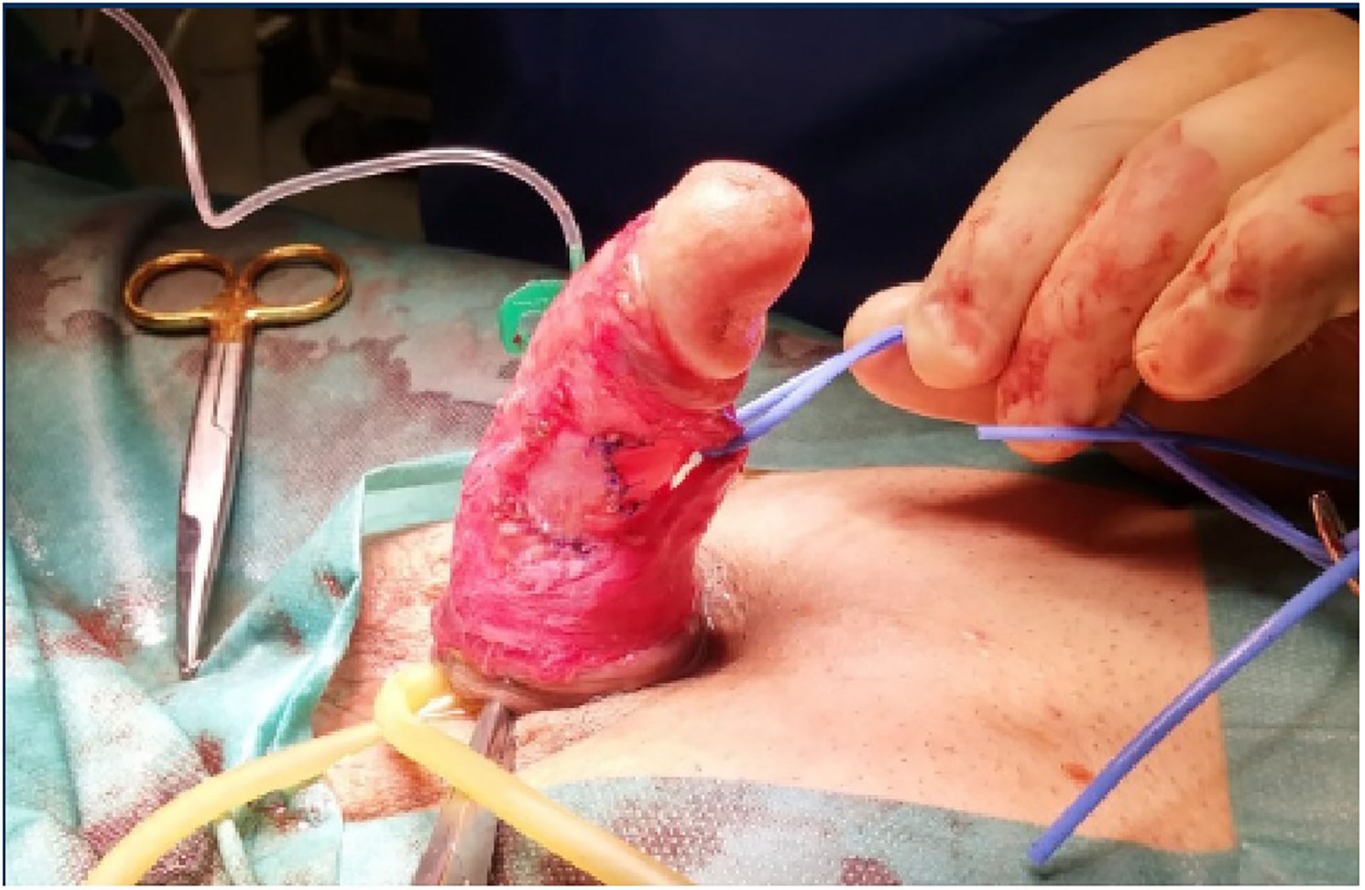
Graft fixation.

In more severe cases, PD patients with a short penis, extensive plaque, severe (>60°), or complex deformities will require a graft procedure. On the side with the concave deformity, an incision is made in the plaque, with or without removal of a portion, and then a graft is used to close it. Depending on the location of the plaque, mobilization of the neurovascular bundle may be required. The patient should always be informed that in these types of procedures there may be the possibility of mobilization of the neurovascular bundle with and this may lead to ED problems (47) (Figures 1–3).

Devine et al. were the first to describe a grafting procedure for correction of the PD curvature (47). Since then, many different graft materials have been used.

**Figure 4:**
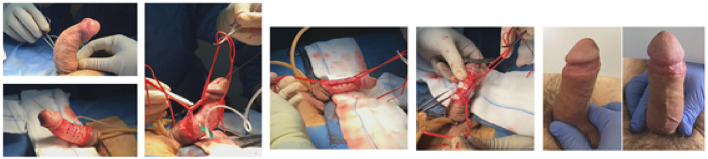
Multiple transverse incisions patched with TACHOSIL®.

The ideal material for grafting does not exist. It must be easy to manipulate and suture, flexible to allow adequate stretching for erection, rigid to prevent aneurysm formation, easily available, cost-effective, and minimal morbidity in its use. The materials currently available can be classified into 4 groups:

- autografts: taken from the patient, such as dermis, saphenous vein, tunica albuginea, fascia lata, and oral mucosa;
- allografts: from a deceased human donor, such as fascia lata, dura mater, and pericardium;
- xenografts: from a donor of a different animal species, such as bovine pericardium, porcine small intestinal submucosa, and matrix of equine collagen;
- Synthetic grafts.
- We have experience applying fibrinogen sponges and human thrombin to correct defects, with favorable results (Figure 4).

The studies were often retrospective, single-center, and did not compare different types of grafts and biomaterials. In conclusion, there is not a single graft that can be recommended for surgeons.

#### Penile Prosthesis Implantation

When the patient has Peyronie's disease associated with erectile dysfunction that is refractory to medical treatment, typically the treatment is based on the placement of a penile prosthesis. Traditionally, formal indications for penile prosthesis insertion in PD patients have been reported. The treatment goals of penile prosthesis implantation are (1) to restore reliable penile rigidity and (2) to provide functional penile straightening. The most used penile prosthesis is the inflatable one, but studies have shown a similar satisfactory response with the use of the malleable model. Small curvatures are usually corrected only with the placement of the prosthesis. There may be a need for penile modeling, done manually after the placement of the prosthesis, consisting of stretching the penis in a contralateral direction to the curvature and maintained for 90 s (48). Complex curvatures that do not respond to the modeling technique must be treated following the previously exposed principles of plication and/or grafting (49).

**Figure 5:**
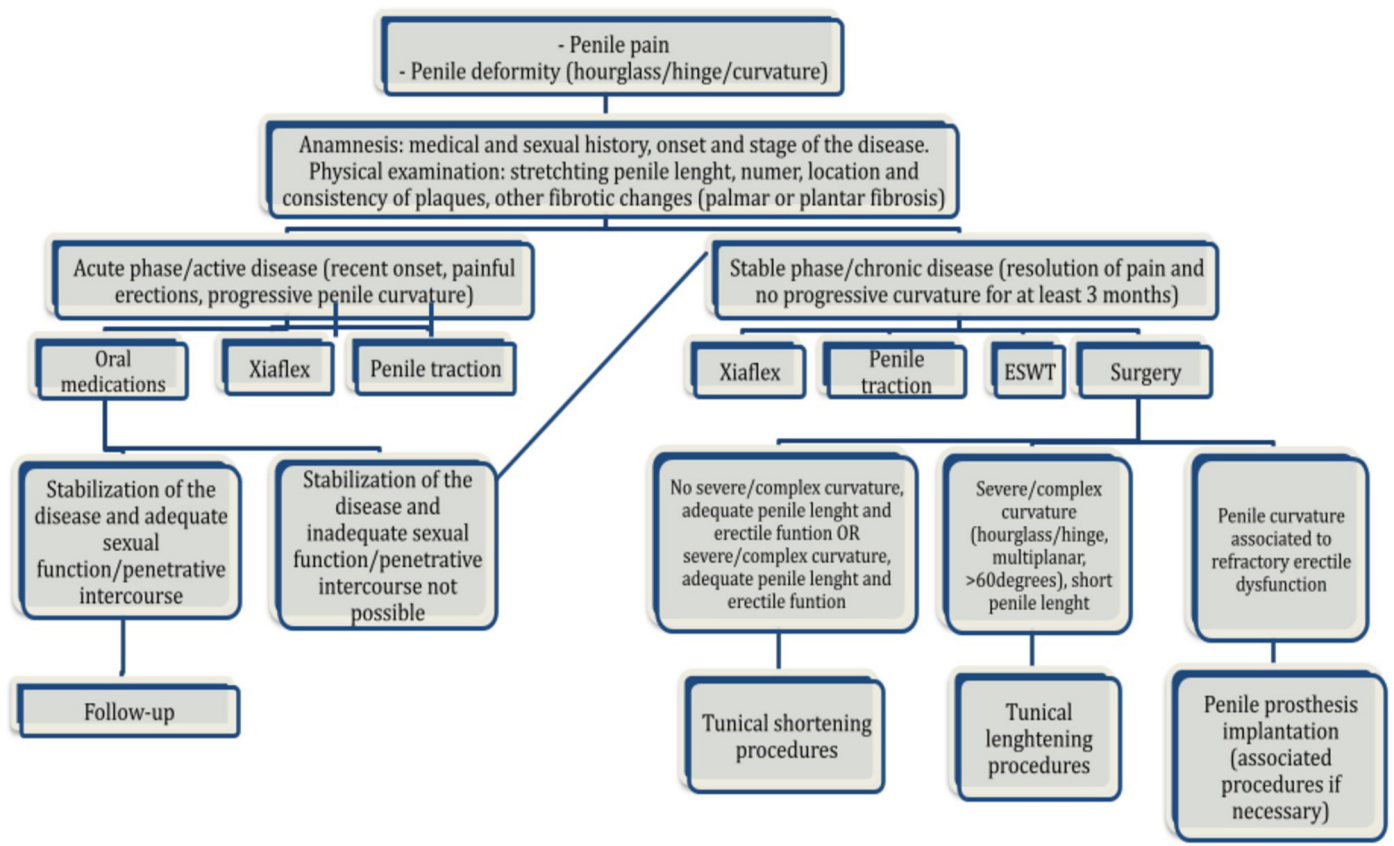
Algorithm for management of Peyronie's disease..

With the advent of Xiaflex (collagenase *Clostridium histolyticum*), a new paradigm of surgical treatment algorithm has been proposed (Figure 5). In 2011, Levine et al. suggested that performing external traction prior to placement of an inflatable penile prosthesis could preserve and possibly increase the erect length of the implant after prosthesis (50). Garaffa et al. reported on the potential need for additional straightening procedures to successfully correct the residual curvature after penile prosthesis implantation alone (51). On the other hand, Mulhall et al. showed that the combined therapy of plate incision with penile prosthesis placement would not be the best option for patients with PE and ED (52). The EAU guidelines on penile curvature recommended a grafting procedure for patients with a degree of curvature is >60° or with a complex curvature, or in patients with a significantly shortened but with a good erectile function (with or without pharmacologic treatment). In patients with erectile dysfunction refractory to pharmacotherapy, the best choice is the insertion of an inflatable penile prosthesis, with or without a concomitant additional penile straightening procedure such as modeling, plication, or even grafting (53). Nonetheless, although all conservative therapeutic options, surgical correction remains the mainstay treatment for this condition (54).

## Conclusions

When treating a patient with Peyronie's disease, the entire context of the repercussions produced in the patient must be considered, since in addition to compromising the psychological part of the patient with anguish due to the changes that are taking place, it causes a progressive aesthetic change, this accompanied by pain and less rigid erections. Several investigations and works have been carried out to stop the acute process of PD without definitive results at present, so surgery continues to be the mainstay of treatment. It should always be taken into consideration that in case of deciding to perform a surgery with plication of the tunica albuginea, this will produce a shortening in the length of the penis and that these should be indicated in case the curvatures are <60°. On the contrary, if the length of the penis is short, if the patient does not want a shortening of the penis, or if the curvature is >60°, the surgical decision can be made to incise the tunica with the placement of grafts, always orienting the patients regarding hourglass or hinge deformities and penises and an increased chance of ED. And in case of severe erectile dysfunction, you can opt for the placement of the penile prosthesis. All of this demonstrates that PD continues to be a great challenge in urological practice, so before proposing a treatment, all the items of each case must be studied together with the patient to carry out a reliable evaluation, understand the benefits, and maintain always presence the risks of each treatment.

## Author Contributions

All authors listed have made a substantial, direct, and intellectual contribution to the work and approved it for publication.

## Conflict of Interest

The authors declare that the research was conducted in the absence of any commercial or financial relationships that could be construed as a potential conflict of interest.

## Publisher's Note

All claims expressed in this article are solely those of the authors and do not necessarily represent those of their affiliated organizations, or those of the publisher, the editors and the reviewers. Any product that may be evaluated in this article, or claim that may be made by its manufacturer, is not guaranteed or endorsed by the publisher.

## References

[1] Chung E Ralph D Kagioglu A Garaffa G Shamsodini A Bivalacqua T. Evidence-based management on Peyronie's disease. J Sex Med. (2016) 13:905–23. 10.1016/j.jsxm.2016.04.062 27215686

[2] Amin S. Herati Alexander W. Pastuszak the genetic basis of peyronie disease: a review. Sexual Med Rev. (2016) 4:85–94. 10.1016/j.sxmr.2015.10.002 27872008 PMC4778255

[3] Gabrielsen JS. Peyronie's disease: is it genetic or not? Transl Androl Urol. (2020) 9:S262–8. 10.21037/tau.2019.10.21 32257867 PMC7108984

[4] Bias WB Nyberg LM Hochberg MC Walsh PC. Peyronie's disease: a newly recognized autosomal-dominant trait. Am J Med Genet. (1982) 12:227. 10.1002/ajmg.1320120213 6213155

[5] Qian A Meals RA Rajfer J Gonzalez-Cadavid NF. Comparison of gene expression profiles between Peyronie's disease and Dupuytren's contracture. Urology. (2004) 64:399. 10.1016/j.urology.2004.04.006 15302515

[6] Devine CJ Somers KD Jordan GH Schlossberg SM. Proposal: trauma as the cause of the Peyronie's lesion. J Urol. (1997) 157:285–290. 10.1016/S0022-5347(01)65361-8 8976281

[7] Jarow JP Lowe FC. Penile trauma: an etiologic factor in Peyronie's disease and erectile dysfunction. J Urol. (1997) 158:1388–90. 10.1016/S0022-5347(01)64222-8 9302127

[8] Rhoden EL Teloken C Ting HY Lucas ML Teodósio da Ros C Ary Vargas Souto C. Prevalence of Peyronie's disease in men over 50-y-old from southern Brazil. Int J Impot Res. (2001) 13:291–3. 10.1038/sj.ijir.3900727 11890516

[9] Sommer F Schwarzer U Wassmer G Bloch W Braun M Klotz T. Epidemiology of Peyronie's disease. Int J Impot Res. (2002) 14:379–83. 10.1038/sj.ijir.3900863 12454689

[10] Stuntz M Perlaky A des Vignes F Kyriakides T Glass D. The prevalence of Peyronie's disease in the United States: a population-based study. PLoS ONE. (2016) 11:e0150157. 10.1371/journal.pone.0150157 26907743 PMC4764365

[11] Cowie CC Rust KF Byrd-Holt DD Gregg EW Ford ES Geiss LS. Prevalence of diabetes and high risk for diabetes using A1C criteria in the U.S. population in 1988-2006. Diabetes Care. (2010) 33:562–568. 10.2337/dc09-1524 20067953 PMC2827508

[12] Bjekic MD Vlajinac HD Sipetic SB Marinkovic JM. Risk factors for Peyronie's disease: a case-control study. BJU Int. (2006) 97:570–4. 10.1111/j.1464-410X.2006.05969.x 16469028

[13] Mulhall JP Schiff J Guhring P. An analysis of the natural history of Peyronie's disease. J Urol. (2006) 175:2115–8. 10.1016/S0022-5347(06)00270-9 16697815

[14] Hellstrom WJ Feldman R Rosen RC Smith T Kaufman G Tursi J. Bother and distress associated with Peyronie's disease: validation of the Peyronie's disease questionnaire. J Urol. (2013) 190:627–34. 10.1016/j.juro.2013.01.090 23376705

[15] Levine LA Greenfield JM. Establishing a standardized evaluation of the man with Peyronie's disease. Int J Impot Res. (2003) 15:S103–12. 10.1038/sj.ijir.3901083 14551586

[16] Özmez A Ortac M Cevik G Akdere H Bakir B Kadioglu A. The effectiveness of 3-D computed tomography in the evaluation of penile deformities in patients with Peyronie's disease: a pilot study. Sexual Med. (2019) 7:311–7. 10.1016/j.esxm.2019.06.009 31324507 PMC6728762

[17] Hauck EW HJackstein N Vosshenrich R Diemer T Schmelz HU Bschleipfer T. Diagnostic value of magnetic resonance imaging in Peryronie's disease – a comparison both with palpation and ultrasound in the evaluation of plaque formation. Eur Urol. (2003) 43:293–300. 10.1016/S0302-2838(03)00003-4 12600434

[18] Yang KK Bennett N. Peryronie's disease and injectable collagenase Clostridium Histolyticum: safety efficacy and improvements in subjective symptoms. Urology. (2016) 94:143–7. 10.1016/j.urology.2016.04.049 27211926

[19] Paulis G Barletta D Turchi P Vitarelli A Dachille G Fabiani A. Efficacy and safety evaluation of pentoxifylline associated with other antioxidants in medical treatment of Peyronie's disease: a case-control study. Res Rep Urol. (2016) 8:1–10. 10.2147/RRU.S97194 26770906 PMC4706125

[20] Alizadeh M Karimi F Fallah MR. Evaluation of verapamil efficacy in Peyronie's disease compared with pentoxifylline. Glob J Health Sci. (2014) 6:23–30. 10.5539/gjhs.v6n7p23 25363175 PMC4796342

[21] Weidner W Hauck EW Schnitker K. Potassium paraaminobenzoate (POTABA) in the treatment of Peyronie's disease: a prospective placebo-controlled randomized study. Eur Urol. (2005) 47:530. 10.1016/j.eururo.2004.12.022 15774254

[22] Ralph D Gonzalez-Cadavid N Mirone V Perovic S Sohn M Usta M. The management of Peyronie's disease: evidence-based 2010 guidelines. J Sex Med. (2010) 7:2359–74. 10.1111/j.1743-6109.2010.01850.x 20497306

[23] Teloken C Rhoden RL Grazziotin TM Ros CT Sogari PR Souto CA. Tamoxifen versus placebo in the treatment of Peyronie's disease. J Urol. (1999) 162:2003. 10.1016/S0022-5347(05)68087-1 10569556

[24] Chung E Deyoung L Brock GB. The role of PDE5 inhibitors in penile septal scar remodeling: assessment of clinical and radiological outcomes. J Sex Med. (2011) 8:1472–1477. 10.1111/j.1743-6109.2011.02217.x 21324095

[25] Palmieri A Imbimbo C Creta M Verze P Fusco F Mirone V. Tadalafil once daily and extracorporeal shock wave therapy in the management of patients with Peyronie's disease and erectile dysfunction: results from a prospective randomized trial. Int J Androl. 2012 35:190–195. 10.1111/j.1365-2605.2011.01226.x 22085227

[26] Müller A Mulhall JP. Peyronie's disease intervention trials: methodological challenges and issues. J Sex Med. (2009) 6:848–61. 10.1111/j.1743-6109.2008.01081.x 19138374

[27] Russell S Steers W McVary KT. Systematic evidence-based analysis of plaque injection therapy for Peyronie's disease. Eur Urol. (2007) 51:640–647. 10.1016/j.eururo.2006.10.042 17092631

[28] Gelbard M Goldstein I Hellstrom WJG McMahon CG Smith T Tursi J. Clinical efficacy safety and tolerability of collagenase clostridium histolyticum in the treatment of Peyronie's disease from 2 large double-blind randomized placebo-controlled phase 3 studies. J Urol. (2013) 190:199–207. 10.1016/j.juro.2013.01.087 23376148

[29] Nguyen HMT Anaissie J DeLay KJ Yafi FA Sikka SC Hellstrom WJG. Safety and efficacy of collagenase clostridium histolyticum in the treatment of acute-phase Peyronie's disease. J Sex Med. (2017) 14:1220e1225. 10.1016/j.jsxm.2017.08.008 28874331

[30] Anaissie J Yafi FA DeLay KJ Traore EJ Sikka SC Hellstrom WJG. Impact of number of cycles of collagenase Clostridium histolyticum on outcomes in patients with Peyronie's disease. Urology. (2016) 100:125–30. 10.1016/j.urology.2016.09.050 27816605

[31] Abdel Raheem A Capece M Kalejaiye O Abdel-Raheem T Falcone M Johnson M. Safety and effectiveness of collagenase Clostridium histolyticum in the treatment of Peyronie's disease using a new modified shortened protocol. BJU Int. (2017) 120:717–23. 10.1111/bju.13932 28612401

[32] Capece M Cocci A Russo G Cito G Gibilei G Cacciamani G. Collagenase clostridium histolyticum for the treatment of Peyronie's disease: a prospective Italian multicentric study. Andrology. (2018) 6:84. 10.1111/andr.12497 29733116

[33] Masterson TA Rezk A Ramasamy R. Characteristics predictive of response to collagenase clostridium histolyticum for Peyronie's disease: a review of the literature. World J Urol. (2019) 38:279–85. 10.1007/s00345-019-02850-3 31250098

[34] Fitch WP Easterling WJ Talbert RL Bordovsky MJ Mosier M. Topical verapamil HCl topical trifluoperazine and topical magnesium sulfate for the treatment of Peyronie's disease–a placebo-controlled pilot study. J Sex Med. (2007) 4:477. 10.1111/j.1743-6109.2006.00417.x 17367443

[35] Martin DJ Badwan K Parker M Mulhall JP. Transdermal application of verapamil gel to the penile shaft fails to infiltrate the tunica albuginea. J Urol. (2002) 168:2483. 10.1016/S0022-5347(05)64173-0 12441945

[36] Riedl CR Sternig P Gallé G Langmann F Vcelar B Vorauer K. Liposomal recombinant human superoxide dismutase for the treatment of Peyronie's disease: a randomized placebo-controlled double-blind prospective clinical study. Eur Urol. (2005) 48:656. 10.1016/j.eururo.2005.04.011 15982798

[37] Gholami SS Gonzalez-Cadavid NF Lin CS Rajfer J Lue TF. Peyronie's disease: a review. J Urol. (2003) 169:1234–41. 10.1097/01.ju.0000053800.62741.fe 12629334

[38] Hatzichristodoulou G Meisner C Gschwend JE Stenzl A Lahme S. Extracorporeal shock wave therapy in Peyronie's disease: results of a placebo-controlled prospective randomized single-blind study. J Sex Med. (2013) 10:2815–21. 10.1111/jsm.12275 23898925

[39] Levine LA Newell M Taylor FL. Penile traction therapy for treatment of Peyronie's disease: a single-center pilot study. J Sex Med. (2008) 5:1468–1473. 10.1111/j.1743-6109.2008.00814.x 18373527

[40] Martínez-Salamanca JI Egui A Moncada I Minaya J Ballesteros CM Del Portillo L. Acute phase Peyronie's disease management with traction device: a nonrandomized prospective controlled trial with ultrasound correlation. J Sex Med. (2014) 11:506–15. 10.1111/jsm.12400 24261900

[41] Ziegelmann M Savage J Toussi A Alom M Yang D Kohler T Trost L. Outcome of a novel penile traction device in men with Peyronie's disease: a randomized single-blind controlled trial. J Urol. (2019) 202:599–610. 10.1097/JU.0000000000000245 30916626

[42] Nesbit RM. Congenital curvature of the phallus: report of three cases with description of corrective operation. J Urol. (2002) 167:1187–8. 10.1016/S0022-5347(02)80380-9 11905897

[43] Garcia-Gomez B Ralph D Levine L Moncada-Iribarren I Djinovic R Albersen M. Grafts for Peyronie's disease: a comprehensive review. Andrology. (2017) 6:117–26. 10.1111/andr.12421 29266877

[44] Yachia D. Modified corporoplasty for the treatment of penile curvature. J Urol. (1990) 143:80–2. 10.1016/S0022-5347(17)39871-3 2294269

[45] Essed E Schroeder FH. New surgical treatment for Peyronie disease. Urology. (1985) 25:582–587. 10.1016/0090-4295(85)90285-7 4012950

[46] Gholami SS Lue TF. Correction of penile curvature using the 16-dot plication technique: a review of 132 patients. J Urol. (2020) 167:2066–2069. 10.1016/S0022-5347(05)65085-9 11956440

[47] Devine CJ Horton CE. Surgical treatment of Peyronie's disease with a dermal graft. J Urol. (1974) 111:44–9. 10.1016/S0022-5347(17)59886-9 4273261

[48] Rahman NU Carrion RE Bochinski D Lue TF. Combined penile plication surgery and insertion of penile prosthesis for severe penile curvature and erectile dysfunction. J Urol. (2004) 171:2346–9. 10.1097/01.ju.0000124042.74905.70 15126818

[49] Wilson SK Delk JR. A new treatment for Peyronie's disease: modeling the penis over an inflatable penile prosthesis. J Urol. (1994) 152:1121–3. 10.1016/S0022-5347(17)32519-3 8072079

[50] Levine LA James R. Traction therapy for men with shortened penis prior to penile prosthesis implantation: a pilot study. J Sexual Med. (2011) 8:2112–2117. 10.1111/j.1743-6109.2011.02285.x 21492409

[51] Garaffa G Minervini A Christopher NA Minhas S Ralph DJ. The management of residual curvature after penile prosthesis implantation in men with Peyronie' disease. BJU Int. (2011) 108:1152–6. 10.1111/j.1464-410X.2010.10023.x 21314814

[52] Mulhall J Anderson M Parker M. A surgical algorithm for men with combined Peyronie's disease and erectile dysfunction: functional and satisfaction outcomes. J Sex Med. (2005) 2:132–8. 10.1111/j.1743-6109.2005.20113.x 16422916

[53] Hatzimouratidis K Eardley I Giuliano F Hatzichristou D Moncada I Salonia A. EAU guidelines on penile curvature. Eur Urol. (2012) 62:543–51. 10.1016/j.eururo.2012.05.040 22658761

[54] Gonzalez-Cadavid NF Rajfer J. Mechanisms of disease: new insights into the cellular and molecular pathology of Peyronie's disease. Nature. (2005) 2:291–7. 10.1038/ncpuro0201 16474811

